# Failure mode and effects analysis: A community practice perspective

**DOI:** 10.1002/acm2.12190

**Published:** 2017-09-25

**Authors:** Bradley W. Schuller, Angi Burns, Elizabeth A. Ceilley, Alan King, Joan LeTourneau, Alexander Markovic, Lynda Sterkel, Brigid Taplin, Jennifer Wanner, Jeffrey M. Albert

**Affiliations:** ^1^ Department of Radiation Oncology McKee Medical Center Banner Health Loveland CO USA

**Keywords:** FMEA, patient safety, process improvement, risk assessment, SRS

## Abstract

**Purpose:**

To report our early experiences with failure mode and effects analysis (FMEA) in a community practice setting.

**Methods:**

The FMEA facilitator received extensive training at the AAPM Summer School. Early efforts focused on department education and emphasized the need for process evaluation in the context of high profile radiation therapy accidents. A multidisciplinary team was assembled with representation from each of the major department disciplines. Stereotactic radiosurgery (SRS) was identified as the most appropriate treatment technique for the first FMEA evaluation, as it is largely self‐contained and has the potential to produce high impact failure modes. Process mapping was completed using breakout sessions, and then compiled into a simple electronic format. Weekly sessions were used to complete the FMEA evaluation. Risk priority number (RPN) values > 100 or severity scores of 9 or 10 were considered high risk. The overall time commitment was also tracked.

**Results:**

The final SRS process map contained 15 major process steps and 183 subprocess steps. Splitting the process map into individual assignments was a successful strategy for our group. The process map was designed to contain enough detail such that another radiation oncology team would be able to perform our procedures. Continuous facilitator involvement helped maintain consistent scoring during FMEA. Practice changes were made responding to the highest RPN scores, and new resulting RPN scores were below our high‐risk threshold. The estimated person‐hour equivalent for project completion was 258 hr.

**Conclusions:**

This report provides important details on the initial steps we took to complete our first FMEA, providing guidance for community practices seeking to incorporate this process into their quality assurance (QA) program. Determining the feasibility of implementing complex QA processes into different practice settings will take on increasing significance as the field of radiation oncology transitions into the new TG‐100 QA paradigm.

## 1 INTRODUCTION

Safe and effective radiation therapy requires the careful coordination of technology and department personnel. Clinical radiotherapy processes contain many discrete choreographed steps that are susceptible to errors. Early incident responses identified the need for quality assurance (QA) measures to identify rare but catastrophic errors.^1^ As a result, a QA methodology was created that narrowly focused on mechanical functionality and dosimetric accuracy.^2^ However, retrospective root‐cause analyses of serious radiotherapy incidents have demonstrated that a large percentage of errors occur because of failures in clinical process.^3^ While device‐specific QA continues to play a critical role, it is now clear that an effective modern quality management program must evaluate the entire clinical process as a complex system prone to human and communication errors. Many QA initiatives have been introduced to help the radiation oncology team avoid process‐related errors that could lead to patient harm (e.g., checklists, record & verify, department quality management committees).^4^, ^5^, ^6^ However, these techniques only consider subsets of complex clinical processes and do not give a quantifiable assessment of overall risk. Radiation oncology has recently adopted process evaluation techniques derived from other industries such as the US Military and product manufacturing,^7^ including process mapping, failure mode and effects analysis (FMEA), and fault tree analysis (FTA). These techniques provide a prospective and quantitative assessment of procedural risk. This allows for a top‐down systems approach to quality management that was not present in past QA methodologies.

Failure mode and effects analysis, as part of a quality management program, has been featured in many recent publications and AAPM initiatives such as the 2013 Summer School and TG‐100.^2^, ^7^ Data have been published on FMEA for stereotactic radiosurgery (SRS),^8^, ^9^, ^10^ stereotactic body radiation therapy (SBRT),^11^, ^12^ tomotherapy,^13^ intraoperative radiation therapy (IORT),^14^ treatment planning,^15^ and dynamic MLC tracking.^16^ These valuable publications primarily come from large institutions where staffing levels may allow for variable amounts of time allocation for projects of this magnitude. Given the time and effort required to complete these projects, they may not be generalizable to smaller institutions. They also focus on the finished FMEA dataset and give little emphasis to the early steps required to make the time and resource efforts a priority in their departments. Failure mode and effects analysis is a resource‐intensive project that may be unattainable for smaller groups, given that a thorough FMEA may take a multidisciplinary group many months to complete.^15^ Ford et al recognized this potential downside to the FMEA process and introduced a streamlined approach intended to shorten the time required to perform FMEA.^17^ However, there are subtleties (e.g., education, department buy‐in, and workflow) to the FMEA process that warrant further consideration when evaluating the practicality of implementing FMEA across different practice settings.

Here, the authors report on our FMEA experience in a small community practice setting. Since clinical process is equally complicated across various practice settings, we were interested in understanding how FMEA might impact resource‐limited clinics. This insight will offer important guidance as sophisticated process evaluation concepts become more mainstream and demand large time commitments. We chose to evaluate our SRS program, as it is largely self‐contained and has the potential to produce failure modes with high clinical impact. Details explaining the specifics of the FMEA technique have been previously well‐described.^2^, ^7^ The focus of the present study is to report the specific initial steps taken to perform an effective FMEA in a small, resource‐limited community practice with the aim of informing development of similar initiatives for other interested groups.

## 2 METHODS

### 2.A Facilitator training

We first identified a process evaluation leader for our department, as FMEA should be led by an appropriately trained facilitator. In general, medical physicists are positioned as department safety authorities and are often selected to lead process evaluation efforts. Our physicist (B.S.) attended the AAPM summer school dedicated to quality and safety in radiotherapy, which provided extensive training on the theory and practice of FMEA, and was thus selected as the facilitator for this project. The summer school transcript^2^ was used as an initial information resource for our FMEA program.

### 2.B Description of meeting activities

- *Initial department education*: Department education was an early focus for us, since we could already anticipate that prioritizing the time and resource effort for this project would be a substantial first hurdle. Process evaluation concepts were introduced to our regional oncology program during a two‐part lunch seminar dedicated to quality and safety in radiation oncology. The main focus of our lunch seminar was to make the somewhat esoteric concepts of quality and safety more tangible to the entire radiation oncology program. Our physics group reviewed a series of high profile radiation oncology accidents, the root‐cause analyses, and the new efforts dedicated to clinical process improvement. This established the link between radiotherapy exposure incidents and process‐related errors for those department members not yet familiar with these concepts. The presentation began with a review of a few patient harm cases presented in the “Radiation Boom” series by Walt Bogdanich published in the *New York Times*.^18^ This was followed by a detailed overview of industries and organizations that have demonstrated a commitment to quality and safety. The various components of process evaluation were then reviewed, and the mechanics of the FMEA recipe were explained. Process mapping, FMEA, and FTA were explained with corresponding examples used as supporting material. The net effect of this education session was a collective consensus that risk evaluation initiatives should be made a priority, and that FMEA can serve as a proactive tool to reduce the risk of patient harm.
- *Team recruitment meetings*: Individual, in‐person meetings were scheduled for the facilitator and FMEA team candidates. This time was used to assess general interest and willingness to participate in the project. Ensuring that each team member was able to commit adequate time and effort helped maintain consistency throughout the project. A final multidisciplinary group of eight team members was identified with representation from each of the following disciplines: dosimetry, radiation therapy (also department administrator), nursing, mid‐level provider, research, front desk/administrative staff, physician, and physics. Given our small department size, this diverse team accounted for a large percentage of our available clinical staff.
- *Initial FMEA team meetings*: We emphasized team education to ensure that each team member had thorough training before starting the FMEA evaluation. The facilitator reviewed the initial seminar presentation to re‐establish the motivation for the project. The facilitator then reviewed the specific FMEA methodology in depth. Process mapping was presented as well as the stepwise recipe needed to perform an FMEA evaluation.
- *Process map meetings*: Our SRS program was identified by team consensus as the most relevant treatment technique for our first FMEA evaluation. The major process steps were determined by team consensus during round‐table discussions. The entire SRS care pathway was considered relevant for this exercise. These meetings required full‐team attendance, and each discipline's input was valued throughout the process. The discussion topics concentrated on understanding how each component of our SRS workflow fit together in sequence. Any differences between team members' recollection of the workflow sequence were resolved, and the process map was not considered complete until final team consensus was reached. Smaller groups were then formed and each process step was delegated to the team member with the most domain expertise. A similar approach to meeting structure was used when the smaller groups met to determine the subprocess steps for their respective areas of expertise. The small groups, each consisting of at least two team members, determined the subprocess steps for each main process branch. Teams were encouraged to provide enough detail such that someone with radiation oncology experience would be able to replicate our procedures. When breakout meetings were completed, the main process branches (including subprocesses) were compiled into a simple linear electronic process map (using Microsoft Publisher) to be used as a complete reference for future meetings and presentations. The completed process map was distributed to each team member for final review and edits. It was also sent for independent evaluation to a small subset of the clinical staff familiar with our SRS program.
- *FMEA meetings*: The FMEA evaluation initially attempted the same strategy used for the process mapping by splitting the FMEA task into smaller assignments to be completed by individuals or small groups. This initial strategy failed, because lack of time and low confidence reduced our ability to complete the FMEA without facilitator guidance. Weekly open sessions with the facilitator were then scheduled, so that team members could attend when their schedule permitted. This strategy allowed us to dedicate protected time to the FMEA evaluation while avoiding conflict with other clinical obligations. No one was required to attend every session, and the attendees determined the process steps that were analyzed in any given session. This allowed us to make progress during every session, while the facilitator played a supportive role and provided consistency with the FMEA ranking metrics. The facilitator started each meeting by displaying the digital process map and a tally of the FMEA evaluations completed to date. The expertise of the day's attendees would direct the choice of which subprocess steps would be evaluated. Our FMEA spreadsheet (Table 2) was completed in real time by the facilitator, and the discussion followed a consistent pattern evaluating each process step for potential failures, causes of failures, and effects of the failure on the patient. By referencing our SRS procedures, we then discussed the current controls in place to prevent each failure mode. Each component of the RPN score [Occurrence (O), Detectability (D), and Severity (S)] was discussed and assigned a 1–10 score. The final RPN score is the product of each component score [RPN = (O)(D)(S)]. Scoring differences between team members would sometimes arise, and they were resolved by choosing the more conservative score. We were able to evaluate an average of 10 failure modes in a 1‐hour meeting. After completing each FMEA evaluation step, we compiled a tally of the highest scoring steps. Any process step that scored greater than 100 or had a severity score equal to 9 or 10 was included in this tally. This decision was based on methods used in other FMEA reports^9^, ^10^ and the desire to maintain efficiency while evaluating the aggregated RPN data. We decided any failure that produced a high severity score was worthy of further analysis regardless of final total score.

### 2.C Post‐FMEA practice changes

Guided by the initial FMEA results, practice changes were made to address our highest RPN scores. New RPN scores were generated to evaluate the impact of these changes.

## 3 RESULTS

### 3.A Time commitment

Our facilitator received training from the 2013 AAPM Summer School dedicated to quality and safety in radiotherapy. This course taught the participants how to perform FMEA, and each lesson was enhanced with active practice sessions that emphasized the classroom concepts. Five days were dedicated to this course for the facilitator. Preparation for the initial department presentation took 3–5 hr. The actual presentation was delivered during a 1‐hour lunch seminar to our regional staff, of which approximately 30 attended. Approximately 30 min were spent with each team member during the team recruitment phase resulting in 4 hours of total meeting time. Team education materials were delivered over two 1‐hour meetings. Topics included a review of the initial presentation materials, a specific review of process mapping techniques, and a primer on FMEA. Eight attendees were present for each of these meetings. Process mapping required approximately 30 hr to complete over a period of approximately 2.5 months. Fifteen major process steps were identified in our SRS workflow, and 1–2 hr were needed to identify the subprocess steps for each major branch. Transcribing the map into electronic format took an additional 3 hours. On average, two people worked on each major process step. Approximately 40 hr were required to complete the FMEA evaluation. On average, three people were present per open session. The total time to completion was 85 session hours plus 5 days of facilitator training. The person‐hour equivalent is difficult to calculate given the nature of our weekly FMEA sessions, but it is estimated at 258 hr (Table 1).

**Table 1:** Time commitment to complete our first FMEA evaluation. Summer school training was not included in the total time estimate since it might not reflect the actual time required for independent self‐training

Task	Session time required	Estimated person‐hours
Project leader training at AAPM Summer School	5 days	40 hr
Initial department education (preparation and delivery)	6 hr	48 hr
Team recruitment	4 hr	8 hr
Team education	2 hr	16 hr
Process mapping (including electronic formatting)	33 hr	66 hr
FMEA evaluation and data collection	40 hr	120 hr
Total (not including AAPM Summer School)	85 hr	258 hr

### 3.B Process map

**Figure 1:**
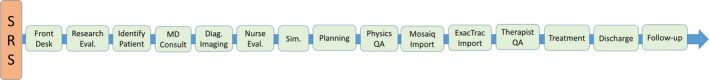
Final process map for our SRS program. Only the major process steps are shown.

**Figure 2:**
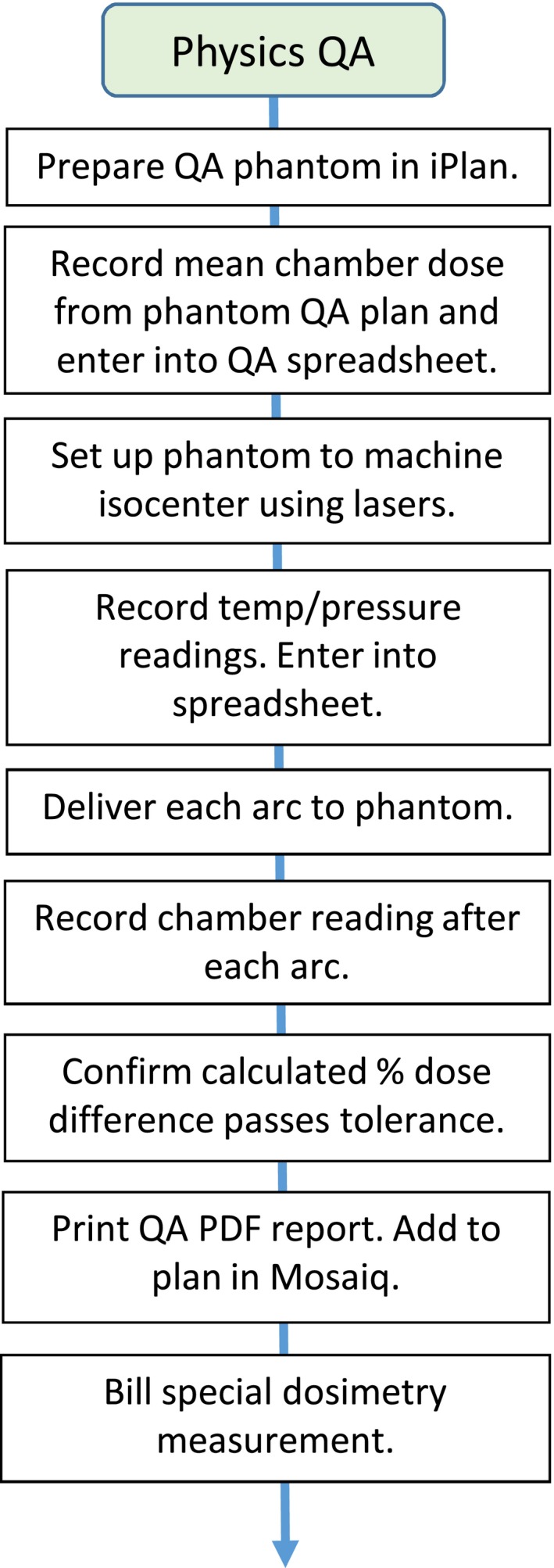
Detail view of the Physics QA process step showing all of the subprocess steps.

Our final SRS process map contained 15 major process steps and 183 subprocess steps. The final process map was built into an electronic format using Microsoft Publisher. We used a simple linear design to aid in evaluation and legibility (Fig. 1). Figure 2 is a detailed view of our physics QA process step.

### 3.C FMEA

**Figure 3:**
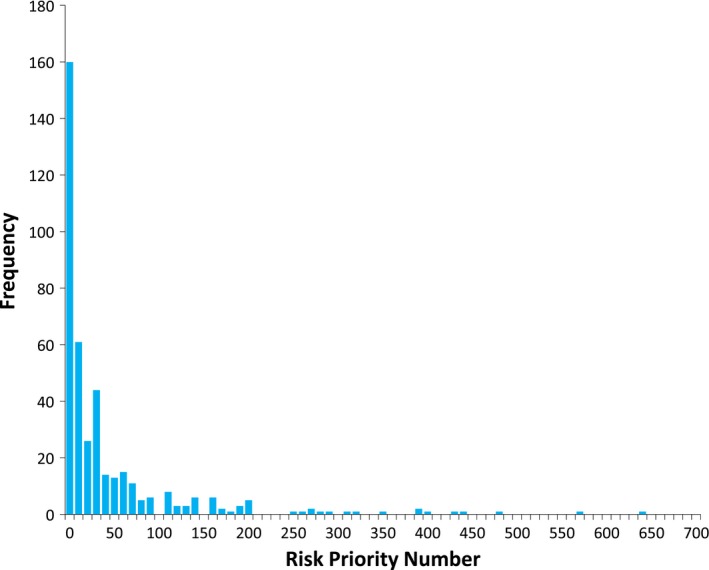
RPN distribution.

The FMEA analysis identified 409 failure modes. Of those, 106 were designated as high‐risk. Table 2 shows our 10 highest scoring failure modes. Figure 3 is a histogram of our FMEA data showing the frequency of occurrence for each RPN score.

**Table 2:** Ten highest scoring failure modes. The “Post‐FMEA Controls” column summarizes the practice changes made following the FMEA evaluation. These changes are indicated in parentheses. New RPN data are also shown based on the practice changes

Major process	Step	Potential failure	Potential cause of failure	Effects of potential failure	Pre‐FMEA controls	(O)ccurrence of cause	(D)etectability of failure	(S)everity of effect	RPN = O×D×S	Post‐FMEA controls (additional controls based on FMEA)	New (O)	New (D)	New (S)	New RPN = O×D×S
Discharge/FU	Schedule follow‐up 3 months after tx.	Not scheduled	Front desk too busy	Patient not being followed by a physician	Memory, department process	9	9	8	648	Memory, department process, (patient discharge instructions, walk patient to front desk if time permits)	3	3	8	72
Discharge/FU	Schedule follow‐up 3 months after tx.	Not scheduled	Front desk does not check orders	Patient not being followed by a physician	Memory, department process	8	9	8	576	Memory, department process, (patient discharge instructions, walk patient to front desk if time permits)	2	3	8	48
Treatment planning	Look for previous treatment	Did not check for previous treatment	Incomplete medical records from another institution	Death	Clinical treatment planning order indicates possible previous xrt, nurse intake form	7	7	10	490	Clinical treatment planning order indicates possible previous xrt, nurse intake form (new item on treatment planning checklist)	3	3	10	90
Treatment planning	Look for previous treatment	Did not check for previous treatment	Incomplete medical records from another institution	Severe adverse event	Clinical treatment planning order indicates possible previous xrt, nurse intake form	7	7	9	441	Clinical treatment planning order indicates possible previous xrt, nurse intake form (new item on treatment planning checklist)	3	3	9	81
Discharge/FU	Schedule follow‐up 3 months after tx.	Not scheduled	Patient leaves	Patient not being followed by a physician	Memory, department process	6	9	8	432	Memory, department process, (patient discharge instructions, walk patient to front desk if time permits)	2	3	8	48
Treatment planning	MD fusion verification	Not verified	Planner assumes MD verified	Unable to plan accurately	SRS planning checklist, treatment delivery checklist	5	9	9	405	SRS planning checklist, treatment delivery checklist, (clarification made to treatment planning checklist to make fusion verification more explicit)	2	2	9	36
Treatment delivery	Physics visual iso check using lasers	Physics did not check	Different physicist did not know	ExacTrac mistranslated laterality and it was not caught	Nothing	5	8	10	400	(New item on treatment delivery checklist)	2	2	10	40
Treatment delivery	Complete SRS delivery checklist	Delivery checklist is not complete	Miscommunication between staff	Inaccurate beam delivery	QCL for dry run, QCL for therapist chart check	4	10	10	400	QCL for dry run, QCL for therapist chart check, (enhanced timeout procedures)	3	3	10	90
Nursing eval	Verify consent signature for contrast	Not verified	Forget/distracted	Severe reaction to contrast	Scheduling process, patient packet, training	4	9	10	360	Scheduling process, patient packet, training, (nurse verifies consent signature at time of IV placement)	2	4	10	
Treatment planning	Check for insurance auth	Did not get insurance auth	Forget/distracted	Patient delay	Physics remembers (weak)	9	9	4	324	Physics remembers, (new item on treatment planning checklist)	3	4	4	48

auth, authorization; eval, evaluation; iso, isocenter; tx, treatment; xrt, radiotherapy.

### 3.D Practice changes following FMEA

Table 2 shows the practice changes we made for our 10 highest scoring failure modes. These changes are indicated in the column, “Post‐FMEA Controls.” New RPN data were generated following the addition of the new practice changes. As a result, each of the original 10 highest RPN scores was reduced to a level below our high‐risk RPN threshold. Many of the severity scores remained at 9 or 10.

## 4 DISCUSSION

This report aims to describe our early FMEA experiences as a small, resource‐limited community practice. The authors believe this perspective will play an important role as the field of radiation oncology transitions into the new TG‐100 paradigm.^7^ As a general community practice, our initial knowledge base should be similar to any other clinic performing FMEA for the first time. The critical first step in starting a process evaluation program is to focus on acquiring department support for new quality improvement concepts that will require significant allocation of time and resources. This started with a formal introduction of the FMEA concept to the entire radiation oncology team, which was done in the context of continuing department education. There are several clear benefits to presenting these concepts in an educational setting. First, it emphasizes the importance of new quality and safety initiatives to the department, which makes it easier to justify time and resource allocation. Further, it demonstrates the interest and dedication of the physics group and other clinical staff in pursuing continuous quality improvement and helps to establish a culture of safety and a culture of learning within the department.

The importance of the facilitator's role has been well‐described in the literature.^2^ It also became clear to us early on that the facilitator is the driving force behind organizing, educating, and focusing the FMEA team. The training that the facilitator receives may be the most important factor leading to the successful completion of an FMEA project. The degree of formal training is dependent on an individual's experience, with TG‐100 being a good starting point. Specific training is currently emphasized by the AAPM through ongoing training sessions at both the chapter and national meeting levels.

**Figure 4:**
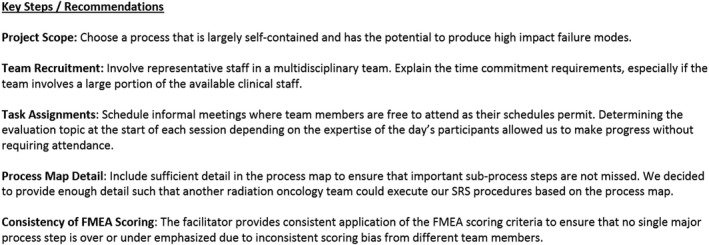
Summary of our insights and recommendations.

Depending on project scope, FMEA can be a large and tedious undertaking. As with any process improvement initiative, FMEA can start slowly when trying it for the first time, and the project's scope may be overwhelming to a small community practice. The following are our direct recommendations/insights based on our experiences working through FMEA for the first time (summarized in Fig. 4). While many of these points are generalizable to any radiation oncology clinic, we found them to be particularly challenging during our FMEA evaluation in a community practice setting:

- *Project scope*: Start with a process that is largely self‐contained, like a special procedure or subset of a larger process. This helps place an upper bound on the project's scope and makes it a reasonable first undertaking. This point is a key recommendation from TG‐100, and it also further recommends that new procedures or resource‐intensive procedures are good candidates for risk evaluation techniques.^7^ We chose SRS for our first evaluation, because it largely stands alone as a separate process in our department and has the potential to produce high impact failure modes.
- *Team recruitment and task assignments*: Depending on department size, the FMEA team may involve the majority of the department's staff in order to provide a comprehensive multidisciplinary perspective. This could make regular team meetings difficult to coordinate, because they will draw considerable resources away from patient care and other clinical duties. This shaped our eventual strategy for completing the evaluation. The early meetings required full attendance as the background and concepts were introduced. These were made a priority, and staff schedules were adjusted accordingly. Our SRS process map was largely built using smaller group assignments, while the facilitator assisted as needed. We tried the same strategy for the FMEA task assignments, but this failed for us. We quickly realized that even though FMEA is easy to understand as a concept, it can be difficult to perform without previous experience or guidance. Instead, we settled on weekly open sessions in a less formal meeting structure where team members were free to attend as their schedules permitted.
- *Process map detail*: Err on the side of more detail in the process map. This ensures that subprocess steps do not contain implied steps that are easily forgotten or missed. The end result is that failure modes are easier to identify. We aimed to provide enough detail such that another radiation oncology team could replicate our SRS procedures based on the process map. This provided a manageable level of detail while also giving us confidence that we were identifying important failure modes. This methodology addresses an obvious weakness in FMEA; that it is difficult to identify all possible failure modes. A similar detailed approach will benefit groups who are new to FMEA, as it minimizes the risk of missing failure modes due to implied steps being hidden in a coarse process map.
- *Consistency of FMEA scoring*: The importance of the facilitator's role is further emphasized during the FMEA evaluation. Consistent application of the FMEA scoring criteria is essential to ensure that no single major process step is over‐ or under‐emphasized due to inconsistent scoring bias from different team members. The facilitator provides an important anchor point that will help maintain consistent scoring during the entirety of the FMEA evaluation.

Despite our best efforts to minimize scoring bias in our FMEA evaluations, it may still exist in our final dataset. Failure mode scoring using FMEA is dependent on the group makeup during each session. Given the nature of our weekly FMEA meetings, only a subset of the entire team was present. Furthermore, there was never an FMEA evaluation meeting where every team member was present. The facilitator was present during each session to help guide the evaluation and minimize scoring bias as much as possible, but potential bias still exists in how the perception of safety was established for each meeting based on the viewpoints of the meeting's participants. If the participants regarded a process step as being generally unsafe, higher FMEA scores would likely result. A different mix of participants could score the same process step differently based on a different perception of safety. Potential evidence of this can be seen in the fact that 3 of the 5 highest FMEA scores came from our patient discharge/follow‐up process step. Nevertheless, we were still able to identify useful failure modes that were previously unknown to our SRS team, especially from the administrative process steps. Changes were made to improve our administrative procedures by creating a more explicit handoff between the nurses and front desk staff, and this had a positive impact for our entire radiation oncology program.

The practice changes we made as a result of our FMEA evaluation focused on introducing redundant checks into a process step. This reduced the probability that the failure's cause will occur (reduce “O”) and increased the likelihood of failure detection (reduce “D”). The practice changes entailed the addition of rather simple items into our workflow, especially on our existing checklists, and subsequent FMEA evaluations based on these changes showed large reductions in RPN scores. Each of our 10 highest RPN scores were reduced following our practice changes and were below our high‐risk RPN threshold (RPN = 100). Severity scores of 9 or 10 still remain since the failure's effect on the patient remains constant.

Our process mapping exercise had an unforeseen positive impact on our SRS program. While our FMEA data certainly improved the safety of our program by revealing previously unknown failure modes, the act of building a process map as a team served to instill a much broader understanding of the complexity of SRS for every team member involved. As a result, each discipline has a more clearly defined role and a better understanding of their relationship to the other process steps. This has led to better overall communication and more efficient planning and treatment workflows.

When compared with other SRS FMEA reports, our dataset is larger in overall number of failure modes, and our highest scoring RPN values also tend to exceed other high scoring data. For example, when compared with Younge et al*,* 9 we identified 409 failure modes as compared to 99 from their study. One hundred six failure modes exceeded our high‐risk threshold, which is higher than their entire failure mode dataset. Our high‐risk inclusion criteria likely explain our large number of high‐risk failure modes. A histogram plot of our FMEA data (Figure 3) shows a distribution that is weighted to RPN scores < 20. The same plot also shows RPN scores that exceed the highest values reported by Younge et al. There are two likely causes for this behavior in our data. First, the predominance of low RPN scores in our dataset may be a result of a highly detailed process map, as much of that detail was not considered high risk by our FMEA team. Second, our high value data may be a result of the scoring bias described above.

The time commitment for this project was rather significant. We suspect that future FMEA evaluations within our program will be quicker due to comfort and familiarity with the steps required to complete the evaluation. Ford et al published an excellent report on a streamlined methodology to quicken the broad‐scope analysis of an entire external beam planning and delivery process.^17^ This was intended to address the question of time allocation and effort for FMEA. Since their report relied on the facilitator's prior experience to create efficiency, their study may not have accurately conveyed the initial effort required for an inexperienced department to start FMEA. It became clear to us early on that facilitator training and expertise are essential for guiding an efficient FMEA. As such, we anticipate that our approach to future FMEA projects will resemble the streamlined methodology described by Ford et al.^17^ Given the nature of our weekly open sessions, it is difficult to calculate an accurate total person‐hour equivalent, but our calculations estimated that 258 person‐hours were required. This is different from Ford's estimate of 75 person‐hours for project completion.^17^ We suspect that this is due to differences in project scope. For example, they identified 62 process steps in their evaluation, whereas our process map contained 183 steps. There may also be a time‐reducing effect due to existing facilitator expertise in the Ford study. We anticipate facilitator experience to have a strong time reduction influence on future FMEA evaluations in our clinic.

To our knowledge, there are no publications from small community practices reporting their experiences with any process evaluation technique. This report provides a perspective on how we performed FMEA for the first time as a small community practice. It was designed to offer important details on the steps we took to progress through our first FMEA evaluation. Based on our experiences and depending on the scope of the chosen clinical process, we estimate that 6–12 months will be required for a clinic to complete their first FMEA. Once the team perfects their FMEA skills, future analyses will likely progress more quickly.

## ACKNOWLEDGMENT

The authors acknowledge the partial support from the Banner Health Risk Management Fund Program.

## CONFLICTS OF INTEREST

The authors declare no conflict of interest.
